# Pediatric nephrologist-intensivist interaction in acute kidney injury

**DOI:** 10.1590/2175-8239-JBN-2022-0158en

**Published:** 2023-04-28

**Authors:** Cassio Rodrigues Ferrari, Carlos Eduardo Lopes, Vera Maria Santoro Belangero

**Affiliations:** 1 Universidade Estadual de Campinas, Faculdade de Ciências Médicas, Departamento de Pediatria, Campinas, SP, Brazil.

**Keywords:** Acute Kidney Injury, Critical Care, Water balance, Injúria Renal Aguda, Cuidados Críticos, Balanço hídrico

## Abstract

**Introduction:**

Acute Kidney Injury (AKI) in the Intensive Care Unit (ICU) have concepts of diagnosis and management have water balance as their main point of evaluation. In our ICU, from 2004 to 2012, the nephrologist’s participation was on demand only; and as of 2013 their participation became continuous in meetings to case discussion. The aim of this study was to establish how an intense nephrologist/intensivist interaction influenced the frequency of dialysis indication, fluid balance and pRIFLE classification during these two observation periods.

**Methods:**

Retrospective study, longitudinal evaluation of all children with AKI undergoing dialysis (2004 to 2016).

**Parameters studied:**

frequency of indication, duration and volume of infusion in the 24 hours preceding dialysis; diuresis and water balance every 8 hours. Non-parametric statistics, p ≤ 0.05.

**Results:**

53 patients (47 before and 6 after 2013). There were no significant differences in the number of hospitalizations or cardiac surgeries between the periods. After 2013, there was a significant decrease in the number of indications for dialysis/year (5.85 vs. 1.5; p = 0.000); infusion volume (p = 0.02), increase in the duration of dialysis (p = 0.002) and improvement in the discrimination of the pRIFLE diuresis component in the AKI development.

**Conclusion:**

Integration between the ICU and pediatric nephrology teams in the routine discussion of cases, critically approaching water balance, was decisive to improve the management of AKI in the ICU.

## RESUMO

**Introdução:**

Os conceitos sobre diagnóstico e conduta da Lesão Renal Aguda (LRA) na Unidade de Terapia Intensiva (UTI) tem como ponto primordial a avaliação do balanço hídrico. Em nossa UTI, de 2004 a 2012, a participação do nefrologista era sob demanda. A partir de 2013, a participação passou a ser contínua em reunião de discussão de casos. O objetivo deste estudo foi determinar como a maior interação nefrologista/intensivista influenciou a frequência de indicação de diálise, no balanço hídrico e na classificação pRIFLE durante esses dois períodos de observação.

**Método:**

Estudo retrospectivo, avaliação longitudinal de todas as crianças com LRA em diálise (2004 a 2016).

**Parâmetros estudados:**

frequência de indicação, tempo de duração e volume de infusão nas 24 horas precedendo a diálise; diurese e balanço hídrico a cada 8 horas. Estatística não paramétrica, p ≤ 0,05. Resultado: 53 pacientes (47 antes e 6 após 2013). Sem diferença significativa no número de internações e nem de cirurgias cardíacas entre os períodos. Após 2013, houve diminuição significativa no número de indicação de diálise/ano (5,85 vs. 1,5; p = 0,000); no volume de infusão (p = 0,02), aumento do tempo de duração da diálise (p = 0,002) e melhora da discriminação do componente diurese do pRIFLE na indicação de LRA. Conclusão: Integração entre equipes de UTI e nefrologia pediátrica na discussão rotineira de casos, abordando criticamente o balanço hídrico, foi determinante para a melhora na conduta da LRA na UTI.

## Introduction

In pediatrics, in the 1960s, Kaplan^1^ presented three cases of acute kidney injury (AKI), all with conservative treatment. In 1971, Dobrin et al.^2^ noted an increase in cases in their clinic (140 children of about eight years of age) and suggested that this was due to factors, such as detection of causes and early signs; development of pediatric nephrologists specialized in nephron-intensive care and improvements in the management of patients with an AKI of one week or more.

In the pediatric group, the most common causes (AKI) in the Intensive Care Unit (ICU) are: postoperative heart surgery, sepsis, hemolytic-uremic syndrome and situations involving cancer therapy^3–5^. In neonates, the most frequent causes include association of the following factors: systemic infection, neonatal asphyxia, low birth weight, prematurity and postoperative heart surgery^6,7,8^.

Until 2004, there were more than 30 definitions of AKI in the literature^9^, ranging from a slight increase in creatinine values to the need for dialysis, preventing the comparison of cases. In 2004, a group of nephrologists and intensivists founded the ADQI (Acute Dialysis Quality Initiative), which proposed a classification of AKI based on a decrease in urinary volume and an increase in serum creatinine values, with the acronym RIFLE (R: Risk; I: injury; F: failure; L: loss of function; E: chronic kidney disease), to characterize the evolution of acute kidney involvement. Serum levels of creatinine and urinary volume were used because they are parameters that are easy to determine in different centers, and which are known to have high sensitivity and specificity for the diagnosis of AKI, in different populations and types of studies^10^. After the RIFLE classification, there were two new AKI classifications proposed: AKIN^11^ and KDIGO^12^.

In 2007, a modification of the RIFLE classification was proposed to adapt it to Pediatrics, with different criteria to define it: the diuresis volume is evaluated every 8 hours and there is no evaluation of the increase in serum creatinine, but of the estimated creatinine clearance^13^. To calculate the estimated clearance, the MDRD (Modification on Diet Renal Disease) formula is not used, but the Schwartz formula (estimated clearance in mL/min per 1.73 m^2^ = k × height (cm)/serum creatinine; k being variable according to age group). As this formula overestimates the measured creatinine clearance, the value of 120 mL/min/1.73m^2^ is used as a reference value for the pRIFLE classification^14^.

The interaction of concepts about the diagnosis and management of AKI in the ICU has as its most important point the assessment of water balance. When positive, the relationship between excess interstitial fluid and acute kidney injury is considered a cause-and-effect potentiator^15^. In addition, positive water balance can lead to difficulty in detecting increased creatinine and delay the diagnosis of AKI^16^.

The importance of diagnosing AKI in critically ill patients is that this complication is associated with increased mortality^17,18,19,20^, length of stay and care costs^21^, being an independent risk factor for death^3,19–21^. Although the frequency of mortality varies, depending on the definition used for AKI and the population studied, it can impact up to 70% of affected patients^3,4,22,23,24,25,26,27^.

The interaction between nephrologists and intensivists in the ICU should complement the diagnosis and adequate management of AKI. The study of this interaction is not enough highlighted in the literature, and only in adult ICU/nephrology populations and teams.

Endre^28^ reports his experience in a general ICU in Australia/New Zealand. After communicating with 7 other nephrologist teams, only the nephrologist participates in the management of intermittent hemodialysis and after discharge from intensive care. The indication of continuous hemodialysis is made by the intensivist, and the contact with the nephrologist is made only at the time of transition to intermittent HD. This model is replicated in so-called “closed” ICUs, to which specialists go only after the intensivist calls, and do not participate in the ICU routine. The participation of the nephrologist in the ICU could help in a better diagnosis of AKI, with a more precise indication of when and how to perform renal replacement therapy and also in its withdrawal, with subsequent follow-up.

Jamme et al.^29^ show in which places the role of the nephrologist is important in an intensive care environment; Initially, to avoid risk factors – nephrotoxicity of medications, fluid overload and correction of medication by clearance –, early diagnosis of AKI and indication of renal replacement therapy, among other factors.

Taking these aspects into account, the objectives of this study are:

- To establish how the nephrologist/intensivist interaction influenced the frequency of dialysis indication and the grading of positive water balance, as well as the components of the pRIFLE score over two observation periods in a pediatric ICU.

## Methods

### Retrospective Study With Longitudinal Evaluation

We included all the children and adolescents submitted to the dialysis procedure over a period of 13 years (2004–2016) within the Pediatric Intensive Care Unit of a tertiary-level hospital, which had 10 beds in the analyzed period. Patients who had previous chronic kidney disease and those who required dialysis within 24 hours of ICU-stay were taken off the study. From 2003 to 2013, the nephrologist’s participation within the ICU occurred on demand from cases with renal involvement. As of 2013, the pediatric nephrologist started to work together with the intensivists. All cases of suspicion or risk of kidney failure were referred to the nephrologist, who began to monitor the cases together. The participation of nephrologists in the weekly meeting of the intensive care unit, with review of cases, also began. There were several sessions of presentation of topics related to hydration and water balance, diagnosis and management of AKI in the ICU, intensive care and septic shock. In these discussions and case reviews, there was a great interaction of knowledge in each area, enabling, in the evolution, to establish protocols, aiming to define conducts for the prevention and early diagnosis of AKI. Aspects relating to the adequacy of doses of nephrotoxic drugs were always highlighted and the prescriptions were adequate to the level of renal function.

The medical records were reviewed to obtain the following data: weight, height, hospitalization diagnosis, water balance, use of diuretics, referral for dialysis, duration of dialysis, hospitalization period, time interval between hospitalization and evaluation of the nephrology. Data related to the pRIFLE were surveyed, having as a reference point the time of dialysis initiation and retroactively as defined in this classification, that is, serum creatinine was analyzed at the time of dialysis indication and 24 hours before the procedure, and urine volume was analyzed every 8 hours, retrospectively for 24 hours after the dialysis procedure.

Data collection for the application of the pRIFLE criterion with analysis of diuresis and estimated clearance was performed on the day of dialysis start and also in the 24 hours preceding the beginning of the dialysis itself. This moment was considered the starting point. As the diuresis criterion is analyzed considering the diuresis every 8 hours, the evaluation was carried out at intervals of eight hours, the first evaluation being considered the one that immediately preceded the start of dialysis, included as day one (D1). Thus, the first 24 hours before the procedure correspond to the first three assessments 1, 2 and 3. Similarly, the estimated clearance was analyzed at the time of dialysis installation, considered as D0 at the start, and D1 before 24 hours.

In the stratification of the pRIFLE criterion classes, regarding diuresis, values above the Risk level, ie >0.5 mL/kg/hour, were considered normal.

The indication for the dialysis procedure was obtained from the medical record. All patients underwent peritoneal dialysis, with access to the peritoneum through placement of a rigid or flexible catheter by the nephropediatric or pediatric surgery team. There were no cases of complications during the procedure or peritonitis.

Creatinine was evaluated using the automated kinetic method, with a modified Jaffé reaction. Estimated clearance was calculated using the Schwartz formula (estimated clearance = k × height (cm)/creatinine (mg/dL), with an estimated normal value of 100^30^.

To calculate the fluid balance, the infused volume was obtained through the controls contained in the nursing record, considering the infused volume (VI) by the sum of all fluids received (“basal serum”, serum for medication, expansions, enteral diet and volume for nasogastric or enteral tube washing). The diuresis volume (DV) was also obtained from the medical records, and all patients included were using a urethral probe to control diuresis. The water balance was calculated every 8 hours, as a result of the following equation: volume of all fluids received minus diuresis. There were no patients with losses other than diuresis. The dose of furosemide used was obtained from the medical prescription, and also computed at 8-hour intervals. The water overload was calculated in 24 hours, with the sum of the water balances in 24 hours, with the following equation: Water overload = 100 × (water balance – diuresis)/diuresis, and presented in percentage, in four classes: negative, between zero and 10%; 10% to 20% and above 20%.

The weight for calculation was obtained from the medical records, reported on the day of admission to the PICU.

The height used to calculate the estimated clearance was obtained from the growth curve of the WHO Child Growth Standards, considering the 50^th^ percentile for all children.

### Statistical Analysis

Numerical data are presented as mean, standard deviation and median. The distribution of the pRIFLE criteria categories for creatinine and diuresis is presented in percentage. The chi-square test was used, considering p ≤ 0.05. Data with frequencies less than 5 were evaluated using Fisher’s exact test.

## Results

### Case Generals

We analyzed 130 patients who required renal replacement therapy during the period. Sixty-two patients were excluded because they had chronic kidney disease with a deficit of renal function prior to admission to the pediatric ICU, and 15 patients because they did not have 24 hours of hospitalization between the indication for the procedure and admission, thus the sample consisted of 53 patients.

The pediatric ICU in the period consisted of 10 beds, with an average admission of 422.2 ± 68.3 (before 2013) and 420.25 ± 83.2 (after 2013) patients per year (Table 1) and an average of 40.33 ± 12.3 (before 2013) and 42.5 ± 10.4 (after 2013) patients undergoing cardiac surgery/year. Both values had no significant differences (p = 0.34 and p = 0.42).

**Table 1:** Clinical data from the case series considering the two observation periods

	Before 2013	After 2013	p
N/year	47/8	6/4	**0.000**
Weight (kg)	8.33 ± 6.54	7.36 ± 3.54	**0.56**
Median (IQ 25–75)	5,0 (4,10–11,00)	6,90 (4,41–10,37)	
Age (m)	20.25 ± 33.63	8.5 ± 7.96	**0.4**
Median (IQ 25–75)	5,0 (2,0–19,00)	5,0 (2,75–16,00)
Number of dialysis/year	5.23	2	**0.002**
Number of hospital stays/year (mean ± SD)	422.2 ± 68.3	430.34 ± 83.2	**0.34**
Number of heart surgeries/year (mean)	44.12	35	**0.42**
Death	32	5	**0.4**

All patients had hypervolemia as an indication. The diagnosis was distributed between cardiac surgery postoperative period (23–43.2%), sepsis (17–35.8%) and other clinical pathologies – liver failure and hemolytic-uremic syndrome (7–20.8%). Compared to the periods, there were no patients in the postoperative period of cardiac surgery after 2013 on RRT. And only 2 patients diagnosed with sepsis and 4 diagnosed with others.

Age, in months, before 2013 was 20.25 ± 33.63 and after 2013 was 8.50 ± 7.96 (p = 0.4) and weight, in kilograms, 8.33 ± 6, 54 before 2013, and 7.36 ± 3.54 after 2013, (p = 0.56), with 54% female. The indication for ICU was secondary to the postoperative period of heart surgery in 43.4%; sepsis in 35.8%; and other situations in 20.8%, with no significant differences between the periods. There was a significant reduction in the number of dialysis procedures/year between the two periods (p = 0.000) (Table 1).

Total infusion volumes (IV) and diuresis volumes (DV), both in mL/Kg/h, for 8-hour periods, for the 24 hours preceding the dialysis procedure, are shown in Table 2.

**Table 2:** Mean, median and standard deviation of the infusion volumes (IV) in mL/kg/h, diuresis volume (DV) in mL/kg/h at time periods 1, 2 and 3 at the time right before the dialysis; values of the entire group and by groups defined by period before 2013 and after

		VI1 mL/kg/h	VI2 mL/kg/h	VI3 mL/kg/h	VI mL/kg/d	VD1 mL/kg/h	VD2 mL/kg/h	VD3 mL/kg/h	VD mL/kg/d
Total	Mean	5.20	4.27	4.51	13.99	1.52	1.34	1.69	4.56
	Median	3.14	3.34	4.02	10.94	1.21	0.95	1.72	4.14
	IQ (25–75)	2,09–5,38	2,51–5,09	2,72–5,85	7,92–17,49	0,65–1,88	0,59–1,73	0,81–2,27	2,51–5,68
		VI1	VI2	VI3	VI/dia	VD1	VD2	VD3	VD/dia
Before 2013	Mean	5.38	4.47	4.65	14.52	1.60	1.46	1.80	4.87
	Median	3.22	3.52	4.28	12.93	1.23	1.20	1.76	4.35
	IQ (25–75)	2,12–5,49	2,70–5,23	2,81–5,89	8,67–18,38	0,67–1,92	0,69–1,87	0,84–2,35	2,88–5,77
After 2013	Mean	3.80	2.66	3.38	9.85	0.88	0.45	0.84	2.18
	Median	2.52	2.29	2.95	7.91	1.05	0.38	0.66	2.10
	IQ (25–75)	1,78–5,87	1,54–3,63	1,56–4,62	5,88–12,91	0,52–1,43	0,00–0,82	0,29–1,43	0,44–3,58
	**p (before × after)**	0.60	0.13	0.20	0.22	0.24	0.06	**0.05**	**0.02**

The 24-hour IV was 13.99 ± 8.86 mL/kg/h, with a median of 10.94 mL/kg/h (IQ – 7.92–17.49) and the 24-hour DV added up to 4 .56 ± 2.82 mL/kg/h, with a median of 4.41 mL/kg/h. Under these conditions, the water balance (WB) calculated every 8 hours was 3.67 ± 6.81 mL/kg/h, with a median of 1.99 mL/kg/h (IQ – 0.31–4.64) in the first period, 2.92 ± 3.12 mL/kg/h, with a median of 2.03 mL/kg/h (IQ – 1.05-3.85) in the second period and 2.75 ± 2.31 mL/kg/h, with a median of 2.61 mL/kg/h (IQ – 1.02-3.99) in the third period. The 24-hour BH was 9.46 ± 8.86 mL/kg/h with a median of 7.18 mL/kg/h. (Tables 2 and 3).

**Table 3:** Values for water balance (WB) mean, median and standard deviation in mL/kg/h, in mL/kg/day and as a percentage of diuresis volume (WB/DV); total dose of furosemide (Furo) in mg/kg/day and the percentage of water overload (WO) on the day before dialysis by patient groups defined by period before 2013 and after

		WB mL/kg/h	WB/DV%	WB mL/kg/d	Furosemide (mg/kg/d)	WB%
Before 2013	Mean	9.50	5.88	228.2	5.40	22.8
N = 47	Median	7.18	0.44	172.3	4.45	17.23
	IQ (25–75)	2,99–13,49	–0,89–4,85	71,76–323,76	1,86–8,18	7,17–32,37
After 2013	Mean	9.06	27.18	217.6	4.70	21.7
N = 06	Median	6.65	0.36	159.6	4.56	15.9
	IQ (25–75)	2,60–15,67	–0,42–68,57	62,46–376,14	3,89–6,13	6,24–37,61
**p**		**0.91**	**0.000**	**0.90**	**0.68**	**0.94**

The water overload in the analyzed period was 22.70 ± 21.26%, with a median of 17.23%. With the distribution of water overload into classes, we found the following distribution: 5.8% without fluid overload, 26.3% with overload lower than 10%, 26.4% between 10 and 20% and 41.5% with greater overload than 20%. (Table 3). All patients used furosemide during the analyzed period, with the doses shown in Table 3.

There was a decrease in the volume of diuresis in volume two (p = 0.06), in volume three (p = 0.05) and in the total volume of diuresis (p = 0.02) after 2013; higher diuresis volume in proportion to water balance in this period 8 (p = 0.000).

The time interval between PICU admission and initiation of renal replacement therapy, the duration of renal replacement therapy and the time interval between initiation of renal replacement therapy and outcome (discharge or death) of patients are presented in the Table 4. Also in these parameters there was a significant increase in the duration of dialysis and the time elapsed between hospitalization and the beginning of the dialysis procedure after 2013. As the participation of the nephrologist was routine, patients had better control of blood volume with more water restriction rigorous (decreased basal serum supply). Longer time for RRT indication may be associated with better control of fluid balance, both data suggesting better control of fluid balance in patients.

**Table 4:** Values for dialysis duration (H) mean, median and standard deviation, and outcome (H) and the time span between hospital stay and dialysis onset (H)

		Dialysis duration (h)	Time between dialysis onset and outcome (h)	Time between hospital stay onset and dialysis onset (h)
Before 2013	X	75.1	24.4	127.80
N = 47	Med	48.0	14.0	66.0
	IQ (25–75)	21,00–116,0	IQ (25–75)	21,00–116,0
After 2013	X	90.3	26.0	149.6
N = 06	Med	48.0	15.0	90.0
	IQ (25–75)	53,75–396,00	16,25–57,75	60,50–530,75
**p**		**0.002**	**0.41**	**0.01**

### PRIFLE Application Result

The distribution frequencies of the criterion “decreased diuresis” of the pRIFLE classification, in the periods of 8 hours, for the 24 hours that preceded the dialysis procedure are shown in Table 5, according to the period studied: before and after 2013.

**Table 5:** Distribution of the “diuresis and creatinine” criteria in the pRIFLE, before and after 2013

Criterion		Period 1 0–8 hours	Period 2 8–16 hours	Period 3 16–24 hours
**Diuresis**	Risk	3	8	2
After 2013	Injury	5	0	2
	Failure	0	0	1
	Without classification	39	39	42
**Diuresis**	Risk	0	1	2
After 2013	Injury	0	2	0
	Failure	2	0	0
	Without classification	4	3	4
**p**	**With × without classification**	**0.33**	**0.06**	**0.07**
		Antes de 2013	Após 2013	**p**
**Creatinine**	Risk	11	0
	Injury	12	1	**0.58**
	Failure	17	5
	Without classification	07	0

The results demonstrate that the diuresis criterion, as a whole, would not be indicative of acute kidney injury in the vast majority of cases, nor does it indicate classification in the Failure category, as expected, since the patients are on renal replacement therapy. Although there were no significant differences in this distribution, there is a trend (with p = 0.06 and p = 0.07) for a lower frequency of unclassified cases after 2013, especially in periods 2 and 3 (Table 5).

As for the estimated creatinine clearance criterion of the pRIFLE classification, at the time of dialysis onset, there were eleven patients (20.8%) in criterion R, thirteen patients (24.5%) in criterion I, twenty-eight two patients (41.5%) in criterion F and seven patients (13.2%) with normal values. In Table 5, these data are presented according to the period studied: before and after 2013. There was no statistical significance in the differences observed in this evaluation, although there was no patient without classification in the second period.

## Discussion

The results presented support the idea that greater integration between nephropediatricians and intensive care specialists brings benefits to patient care in the Intensive Care Unit. There was an important change in the dynamics of the PICU during the period investigated. In 2012, the nephropediatrics team became part of the intensive care case discussions, assimilating important concepts about the critically ill patients, at the same time that the intensivist began to pay more attention to the patients’ water supply. Riley et al.^31^, in an article from 2018, presented data on changes that occurred in their service, among them the presence of the nephropediatrics team to the intensive care environment. The results found were a 12% increase in CRRT, but with a slight decrease in fluid overload (17% to 14%). In our clinic, we found a significant decrease in the number of indications for dialysis, although patients in need of renal replacement therapy (very few) still had significant hypervolemia. 

When analyzing the different parameters between the two periods, there was no difference in the average number of hospitalizations or in the number of cardiac surgeries per year, as well as in the general characteristics of the patients, in the intensity of the fluid overload or in the doses and frequency of diuretic use. The big difference lies in the significant decrease in the number of indications for dialysis. In our view, the main determinant of this encounter is due to the early detection of risk factors for AKI and prevention of hypervolemia, preventing patients from progressing to the need for renal replacement therapy. This fact is also detected in the number of patients in the postoperative period of heart surgery: after 2013, there were no cases of patients without creatinine criteria who required renal replacement therapy.

The results of this study demonstrated that there was a marked change in several parameters, notably in water balance. The diuresis criterion was not able to detect acute kidney injury, especially pre-2013, with 82% to 89% of patients not classified in the three periods analyzed before the start of renal replacement therapy. After 2013, this assessment is impaired, as there is a significant reduction in the number of patients (n = 6). Even so, the percentage of patients without a diagnosis is around 60%, here also emphasizing the effect of volume overload, acting as a confounder for the application of pRIFLE. This result differs from that found in the literature. Koeze et al.^32^, in a study comparing the pRIFLE, AKIN and KDIGO criteria, demonstrated that, when comparing diuresis and creatinine criteria, the diuresis criterion detects renal injury an average of 11 hours before and that most patients will not also reach the creatinine criterion. The AWARE study (2017) showed that the diuresis criterion is more sensitive for the diagnosis of AKI, with 67.2% presenting only the diuresis criterion^33^.

When analyzing the creatinine criterion, we have a better detection with only three patients showing no change in this criterion. The AWARE^33^ shows that the vast majority of patients are on stage 3 KDIGO (equivalent to stage F) at the time of renal replacement therapy.

The results of this study should be considered taking into account that there were some non-ideal conditions for a scientific study: the retrospective aspect is associated with insurmountable biases. The lack of initial stature was an aspect that required a strategy that was not free from criticism. However, the collection of other data was very careful and took into account the notes of the patient’s clinical controls, which are accurately prepared at regular intervals by the nursing service, bringing confidence to the interpretation of the different clinical parameters used. On the other hand, there was an important difference in the observation times periods, and in the number of cases with an indication for dialysis, the first being much longer than the second. But this inconvenience is minimized by the fact that the number of patients/year admitted to the ICU was similar throughout the period, making the differences in dialysis indication more convincing. Although not included in the study, annual statistics have shown an identical profile to period 2, up to the present time (data not shown). The determination of the ICU severity index only became routine from 2016 onwards, making it impossible to compare it between periods. However, the ICU where the study was carried out is located in a teaching hospital, with a history of more than 30 years of operation, with a very stable population demand, which may suggest that there have not been important changes in the profile of the patients treated.

## Conclusion

The integration between ICU and pediatric nephrology teams in the routine discussion of cases with the mutual understanding of the systemic inflammatory response, the evolution of the renal lesion to acute and the repercussion of positive water balance significantly reduced the number of patients requiring renal replacement therapy. The analysis of the pRIFLE parameters in the two time periods showed an improvement in the characterization of the criteria indicative of renal failure (especially the diuresis criterion) after the greatest nephrointensivist interaction.
